# Use of glucometer and fasting blood glucose as screening tools for diabetes mellitus type 2 and glycated haemoglobin as clinical reference in rural community primary care settings of a middle income country

**DOI:** 10.1186/1471-2458-12-349

**Published:** 2012-05-14

**Authors:** Benja Muktabhant, Pattara Sanchaisuriya, Pongdech Sarakarn, Worawitaya Tawityanon, Mantana Trakulwong, Songsri Worawat, Frank P Schelp

**Affiliations:** 1 Department of Nutrition, Faculty of Public Health, Khon Kaen University, 123 Mitharaphap Road, Khon Kaen, 40002, Thailand; 2 Department of Biostatistics and Demography, Faculty of Public Health, Khon Kaen University, 123 Mitharaphap Road, Khon Kaen, 40002, Thailand; 3 Na Klang Hospital, Na Klang District, Nong Bua Lamphu Province, Thailand; 4 Faculty of Public Health, Khon Kaen University, 123 Mitharaphap Road, Khon Kaen, 40002, Thailand

**Keywords:** Diabetes mellitus, Screening, HbA1c, Middle income country

## Abstract

**Background:**

Thailand is considered to be a middle income country, and to control and prevent type 2 diabetes mellitus (T2DM) is one of the main concerns of the Thai Ministry of Public Health (MoPH). Screening for T2DM and care for T2DM patients has been integrated into the primary health care system, especially in rural areas. The intention of this investigation is to link public health research at the academic level with the local health authorities of a district of a north-eastern province of the country.

**Methods:**

Epidemiological methods were applied to validate the screening tools fasting capillary blood glucose (CBG), measured by glucometer and venous blood for the determination of plasma glucose (VPG), used for screening for T2DM among asymptomatic villagers. For assessing the validity of these two methods glycated haemoglobin (HbA1c) values were determined and used as the ‘clinical reference’.

**Results:**

All together 669 villagers were investigated. Determinations of CBG and VPG resulted in suspected T2DM cases, with 7.3% when assessed by CBG and 6.4% by VPG using a cutoff point of 7 mmol/L (126 mg/dl). Taking HbA1c determinations with a cutoff point of 7% into account, the proportion of T2DM suspected participants increased to 10.4%. By estimating sensitivity, specificity and the positive predictive value of CBG and VPG against the ‘clinical reference’ of HbA1c, sensitivity below 50% for both screening methods has been observed. The positive predictive value was determined to be 58.5% for CBG and 56.8% for VPG. The specificity of the two screening tests was over 96%.

**Conclusions:**

The low sensitivity indicates that using fasting CBG or VPG as a screening tool in the field results in a high proportion of diseased individuals remaining undetected. The equally low positive predictive values (below 60%) indicate a high working load for the curative sector in investigating suspected T2DM cases to determine whether they are truly diseased or false positive cases according to the screening method. Further implications of the results and the controversial discussion related to the use of HbA1c as clinical evidence for suffering from T2DM are also discussed.

## Background

It has been estimated that the proportion of diabetes mellitus among adults between the ages of 20 and 79 was 6.4% in the year 2010 and will increase to 7.7% in 2030 world wide [1]. Sixty-nine percent of the increase is expected to be in the so-called developing countries and 20% in developed countries. The morbidity and mortality pattern of the developed countries is now highly prevalent in the low and middle income countries as well, forcing the health delivery systems of these countries to face the challenge of coping with the double burden of infectious and chronic diseases [2]. One of the major attempts to improve the health of the population especially in the low and middle income countries has been the primary health care attempt inaugurated at the Alma Ata meeting over 30 years ago [3]. The focus during the foregoing decades had been primarily laid on infectious diseases and mother and child health care (MCH), and Thailand has performed quite well in controlling a number of important infectious diseases and significantly improved MCH care [4,5]. However, there has been an increase in the morbidity of chronic diseases among the Thai population, in part due to the so-called nutritional transition [6]. Intake of energy above requirements in connection with physical inactivity has resulted in over nutrition and obesity of large portions of the population, accompanied by the rapid development of diabetes mellitus type 2 (T2DM). In Thailand, T2DM ranks as number 8 for males and as number 2 for females in the list of major diseases contributing to disability adjusted life years (DALY) [7]. Efforts to reduce the spread of chronic diseases including primary and secondary preventive measures nowadays have a high priority and have been integrated into primary health care by the Ministry of Public Health (MoPH), focusing especially on T2DM.

In initiating screening for T2DM in the rural areas the Ministry of Public Health (MoPH) has followed the recommendation of the International Diabetes Federation (IDF) which pointed out in a recent review the overall benefits of screening and concluded that ‘*individual countries should aim to develop and evaluate cost-effective methods, setting up specific diabetes risk identification and prevention strategies based on available resources*’ [8].

In order to assist the efforts of the Ministry, the authors decided to launch research activities in a rural area of a province in the north-eastern part of the country (Nong Bua Lamphu Province) in co-operation with the local health administration at the district and sub-district levels. The Na Klang District of Nong Bua Lamphu Province, among other areas in Thailand, has been selected as the site of a pilot project by the MoPH for an initiative to assure the quality of primary health care especially in the field of non-communicable diseases, focusing on the occurrence of some aspects of the metabolic syndrome at the community level. It was not intended to launch a strictly academic driven study but to investigate a screening attempt on population basis undertaken with limited resources by the district and sub-district health officials. The results might cause the health authorities to reconsider some of the measures applied and by this improve the effectiveness of the measures.

The specific objective of this study is to evaluate the screening attempts for T2DM undertaken by the local health authorities and to validate the screening tools namely fasting capillary blood glucose (CBG) measurement by glucometer kits and fasting plasma glucose (VPG) determination, verified by glycated haemoglobin (HbA1c) as the clinical reference.

## Methods

### Area of investigation

Nong Bua Lamphu Province is one province of the Northeast of Thailand. The overall population density is estimated to be 125 inhabitants/km^2^. The area is subdivided into 6 districts with 59 sub-districts and 636 villages. Na Klang, the district selected for this study, has an estimated total population of 91,000 inhabitants living in 8 sub-districts with a total of 131 villages served by 13 primary care units (PCUs). From each PCU approximately 60 villagers participated; the total number of participants tested was 669.

### Routine district screening process

The attempts to control T2DM through screening are a collaboration with the local health officials of the district hospital and the staff of the PCUs. Screening for T2DM is targeted towards the adult population aged 35 years and older. Village health volunteers (VHVs) together with the staff of the PCUs encourage the villagers in the communities to participate in the screening program. The district hospital serves as a referral institution to verify screening results and assist in the treatment of the disease and its complications.

The routine screening process undertaken by the district health staff involves asking the target group to fast overnight and then taking one CBG sample from the finger tip to assess glucose by glucometer (Accu Check Advantage®, Roche) and measuring blood pressure. Individuals found to have fasting blood glucose values of ≥7.0 mmol/L (≥126 mg/dl) are referred to the community hospital for verification of the screening results. Verification is done by analysis of one sample of fasting VPG levels using the glucose oxidase method (Glucose Oxidase Data Pro., Thermo Scientific®). In cases where the verification via VPG produces a result of ≥7.0 mmol/L (≥126 mg/dl), the patient is registered as a T2DM patient and cared for by the T2DM clinic set up by the district hospital. However, individuals found, through the CBG screening process, to have glucose values between 6.1 mmol/L (110 mg/dl) and 6.9 mmol/L (124 mg/dl) are asked to come to the PCU after approximately 6 months to be rechecked, using CBG again.

### The validation of the screening process

This study aimed to test the fasting CBG and VPG used routinely against the reference value of HbA1c. For that purpose, the routine method described above was followed in principle, but amended slightly for the participants of this study.

All of the participating 13 PCUs were asked to recruit approximately 60 villagers who had no previous T2DM diagnosis. On the day screening took place in the respective community either the staff of the PCUs or the VHVs took blood from the fingertip for CBG determination by the glucometer kits. Immediately after that the staff of the PCU drew 3 ml of venous blood for laboratory analysis. Blood was separated into two tubes: 1 ml into a tube containing sodium fluoride for fasting blood sugar and 2 ml into an EDTA tube for HbA1c determination. Tubes were stored not more than two hours before being transferred to the laboratory. Plasma for fasting blood sugar determinations was obtained by centrifugation and stored at +4^0^C until being analyzed using the glucose oxidase method within two weeks. As the ultimate diagnostic tool for the diagnosis of T2DM, glycated hemoglobin (HbA1c) was determined by turbidimetric inhibition immunoassay (Kenolab/T series; Thermo Scientific®), at the laboratory of the district hospital. All HbA1c determinations were done with only one newly purchased equipment. Calibration and quality control measures were strictly followed as recommended by the supplier of the equipment. Sex and age of the participants were recorded as well.

### Data processing and statistical methods used

The data entered into an MS Excel spreadsheet by the health officials of the district hospital of the participating district were rearranged and revised by the staff of the Department of Nutrition of the Faculty of Public Health, Khon Kaen University. The data were then transferred from the Excel program into a MINITAB Version 12 spreadsheet for further statistical evaluation. Ordinary descriptive statistics were used for evaluating the data. Sensitivity, specificity, positive (PPV) and negative predictive values (NPV) were calculated and the SPSS statistical program Version 17 was used for modeling the receiver operating characteristics (ROC curves).

### Ethical approval

Ethical approval was obtained from the ethical committee of the Khon Kaen University (HE 532243).

## Results

### Number, age and proportion of suspected DM cases

Alltogether 669 villagers from 13 PCUs participated in the project. The ratio of females to males was 2.3, with the average age of males being 53 and that of females being significantly different at 48. The results of the number and percentages of individuals suspected to have DM is given in Table 1. Determinations of CBG and the VPG resulted in similar percentages of suspected T2DM cases, being 7.3% for the CBG and 6.4% for VPG. By taking HbA1c determination with a cutoff point of 7% into account, the percentage of suspected T2DM cases increases to 10.4%, a significant increase over the values obtained by measuring fasting blood glucose using a cutoff point of 7 mmol/L (126 mg/dl).

**Table 1:** Number and percentage of villagers suspected to suffer from diabetes mellitus type 2

**Variables**	**N** **Total**	N* Suspected DM	Percent*	95% C.I.**
**CBG**	669	49	7.3	5.5 – 9.6
**VPG**	669	43	6.4	4.7 – 8.6
**HbA1c**	656	68	10.4	8.2 – 13.0

*Number and proportion of villagers with fasting blood glucose levels of ≥7.0 mmol/L determined by glucometer and laboratory method as well as ≥7% for HbA1c.

**C.I. Confidence interval.

### Validity of CBG and VPG results as screening tools versus each other and HbA1c

The CBG determinations as screening tool and VPG as reference with a cutoff point of 7 mmol/L (126 mg/dl), resulted in a sensitivity of 81.4% and specificity of 97.8% and a PPV of 71.4% (Table 2). When testing the values obtained by CBG and VPG as screening method and HbA1c values at a cutoff point of 7% as clinical reference, sensitivity decreased below 50% for both methods, 45.6% for CBG and 39.7% for VPG. Consequently, the PPV also decreased to 58.5% for CBG and 56.8% for VPG. Specificity of all the tests was over 96%.

**Table 2:** Sensitivity and specificity as well as positive- and negative predictive values

**Screening variable**	Clinical Reference *	**N** **Total**	**Sensitivity**	**Specificity**	PPV**	NPV**
**CBG**	VPG	669	35/43 (81.4%)	612/626 (97.8%)	35/49 (71.4%)	612/620 (98.7%)
**CBG**	HbA1c	656	31/68 (45.6%)	566/588 (96.3%)	31/53 (58.5%)	566/603 (93.9%)
**VPG**	HbA1c	645	25/63 (39.7%)	563/582 (96.7%)	25/44 (56.8%)	563/601 (93.7%)

*VPG ≥7.0 mmol/L and HbA1c ≥7%.

**PPV Positive predictive value, *NPV* Negative predictive value.

### Receiver operating characteristics (ROC) curves

**Figure 1:**
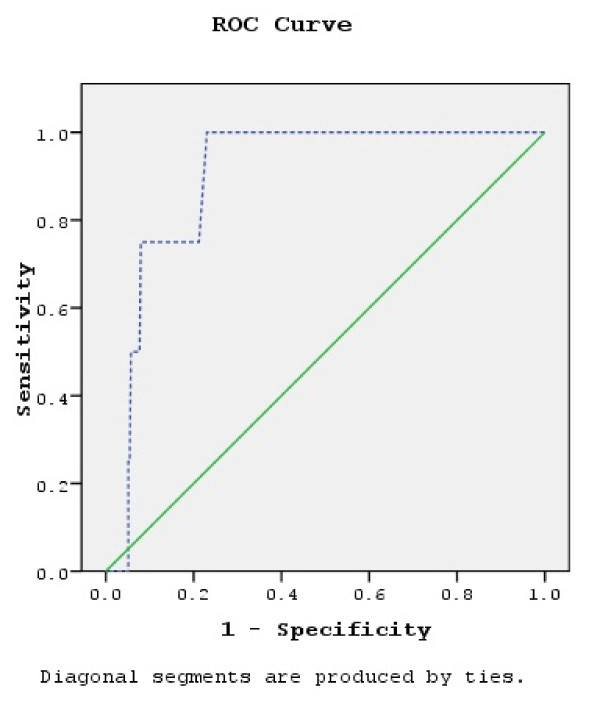
**ROC curve with CBG values as screening variable and VPG as reference (positive actual state 7.0 mmol/L).** Original calculation has been done with glucose concentration measured in mg/dl

**Figure 2:**
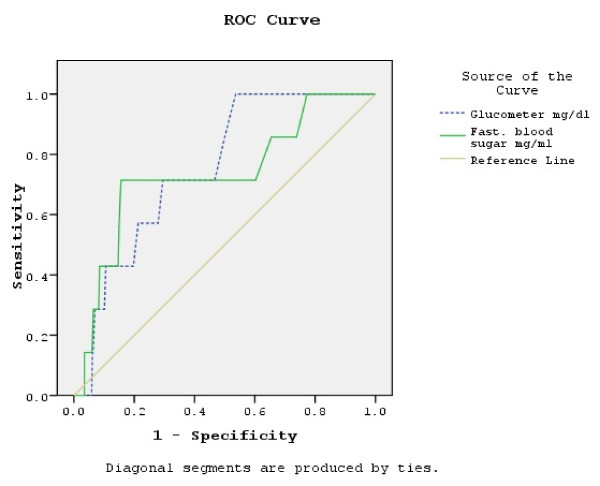
**ROC curve with CBG and VPG as screening variables and HBA1c as reference (positive actual state 7%).** Original calculation has been done with glucose concentration measured in mg/dl

The ROC curve with CBG values as the screening variable and VPG values as the reference (positive actual state 7.0 mmol/L (126 mg/dl)) is shown in Figure 1 and the ROC curves with CBG and VPG as the screening variables and HbA1c as the reference (positive actual state 7%) are provided in Figure 2. Statistical indicators for the interpretation of the three ROC curves are displayed in Table 3. The largest area under these three ROC curves is 0.898, indicating that statistically the best combination is VPG as reference with the CBG results as the screening tool. The smaller area under the curve for the case where HbA1c is the reference and VPG is the screening tool is 0.733. Comparing the lower and upper bounds of the 95% C.I., CBG as a screening tool has a more narrow range (0.620 to 0.888) in comparison to VPG (0.529 to 0.938), taking HbA1c as reference. According to the result of the ROC curves, optimal results would be achieved with a cutoff point of 5.6 mmol/L (101 mg/dl) taking CBG as the screening tool and the VPG as the reference. Sensitivity in this case would be 75% and specificity 78.8%. Optimal cutoff point for CBG would be 5.03 mmol/L (90.6 mg/dl) when taking HbA1c as the reference (with a cutoff point of 7%). Sensitivity would be then 85.7% and specificity 50%. Using VPG as the screening tool, the optimal cutoff point would be 4.6 mmol/L (82.9 mg/dl) when HbA1c is the reference (cutoff point of 7%). The sensitivity in this case would also be 85.7%, but the specificity would decrease to 26.2%.

**Table 3:** Interpretation of ROC curves

	**VPG (Ref.) vs CBG**	**HbA1c (Ref.) vs. CBG**	**HbA1c (Ref.) vs. VPG**
**Positive state for ROC curve**	7.0 mmol/L	7%	7%
**No. positive**	4	7	7
**No. negative**	665	634	634
**Area under curve**	0.898	0.754	0.733
**Std.Err.**	0.036	0.068	0.104
**Asympt. Sig.**	0.006	0.021	0.033
**95% CI** ** Lower bound** ** Upper bound**	0.827 0.970	0.620 0.888	0.529 0.938
**Optimal test result for**	5.6	5.03	4.6
**Sensitivity**	75.0%	85.7%	85.7%
**Specificity**	100 – 21.2%	100 – 50.0%	100 – 73.8%

## Discussion

The population of the study area is genetically quite uniform and no different ethnic groups are living there. Screening for T2DM takes place in a totally different environment in high income countries compared to countries in the category of low or middle income, such as Thailand. So-called primary care in high income countries usually is a ‘passive’ service provided by the ambulatory care section of the health delivery system, in that individuals are encouraged to see their general practitioners in their clinic and they only undergo a test according to the decision of the particular clinician they consult. In Thailand the MoPH has initiated a more active screening effort in rural areas, where the general population is being approached by VHVs to assemble at a central spot within their community after a nights fasting to be screened by the staff of the PCU in charge of the village using glucometer kits. Whatever the MoPH advocates has to be easy to perform, must be time and cost effective, and care has to be taken not to overload the clinical arm of the health delivery system, such as district and provincial hospitals, with a heavy load of false positive cases indicated by a low PPV.

It is not feasible for the understaffed and overworked health officials at the sub-district level to take more than one CBG sample and come back to the same village after about one week and take another sample. Also supervision of the staff by their superiors from the provincial level is not possible when doubling the work. Also, to actually relate the result from the first and the second sample to one and the same individual needs accuracy which cannot be always expected from a public health nurse with a two years training let alone from a VHV. Similarly, to conduct an oral glucose tolerance test (OGTT) on a population basis is beyond feasibility. One round in one district might involve testing more than 7,000 individuals in 119 villages.

This study attempted to follow the screening procedures of the Thai health delivery service of the participating district as much as possible in order to evaluate its achievements but also its constraints. According to the study plan it was not intended to draw a representative sample of adults from the district. Still, the proportion of individuals found to have fasting blood glucose concentrations of 7 mmol/L (126 mg/dl) and above was similar to the T2DM prevalence estimated in nationwide surveys, which is reported to be 9.6%, with 4.8% newly diagnosed cases in 2000 [9] and 6.7% in 2004 [10]. The latter estimate was age standardized based on the Thai national population. In 1995 a representative sample of three districts of a neighboring province (Khon Kaen) of the study area detected a prevalence of T2DM of 11.9% with a 95% C.I. of 8.4 to 16.5 by using the OGTT [11]. This figure compares well to the proportion of individuals found in this study to have HbA1c levels of 7% and over, which is 10.4%.

Since this study is restricted to reporting the validity of screening tests to detect T2DM diseased patients, the proportion of Impaired Fasting Glucose (IFG), in the range of 6.1 mmol/L (101 mg/dl) to 6.9 mmol/L (124 mg/dl), has not been reported. As outlined in the methodology section, in the real field situation individuals found to have fasting blood glucose levels of 6.1 mmol/L (101 mg/dl) to 6.9 mmol/L (124 mg/dl) are advised by the staff to come to the nearest PCU in approximately 6 months to be rechecked, at which time it may be assumed that they will have a fasting blood sugar level over 7 mmol/L (126 mg/dl) if they are indeed early T2DM cases. Since it is estimated that half of all individuals having T2DM in Thailand remain undetected [9], the aim of the health authorities at this time is to concentrate clinical attention on those being found to already have T2DM. Using fasting blood glucose with a cutoff point of 7.0 mmol/L (126 mg/dl) as a valid test for the diagnosis of T2DM is recommended by the World Health Organization (WHO) [12]. Using CBG as a tool for screening and VPG determinations as a final diagnosis results in a sensitivity of over 80%, a specificity of over 95% and a PPV of 70%. Using CBG as a screening indicator against VPG as standard, the results as far as sensitivity, specificity and PPV are concerned seems to be acceptable. According to the ROC curve of this investigation the optimal cutoff point would be 5.6 mmol/L (101 mg/dl), which is in accordance with the findings of Nitiyanant et al. [13], who recommended a cutoff point between 5.6 mmol/L (101 mg/dl) and 6.0 mmol/L (108 mg/dl) for the Thai population.

When CBG values are used as a screening tool and VPG as a reference, less than 20% of those screened remained with undetected T2DM. However, when selecting HbA1c as the reference and CBG as the screening tool, 54.4% of suspected T2DM cases are not identified and the proportion of false negative results increases to 41.5%. In the case where VPG is the screening tool and HbA1c is the reference, 60.3% of T2DM cases are not identified and the curative center is unnecessarily burdened with 43.2% of the cases, which are false positives.

HbA1c was recommended as a diagnostic tool as far back as 1976 by Koenig et al., [14,15] and its use has increased over time in clinical settings in high income countries. For example a survey undertaken in Ontario (Canada) found that in 2005 HbA1c were determined in approximately 500,000 individuals without diabetes and for more than 480,000 cases with diagnosed diabetes [16]. In the same year, 37% of the Ontario adults without T2DM were tested by serum blood glucose and less than 1% of clinicians used the OGTT. The controversial discussion about the application of HbA1c for either screening or clinical diagnosis intensified after the American Diabetes Association (ADA) and the European Association for the Study of Diabetes recommended the use of HbA1c to diagnose T2DM [17]. That recommendation was included in the ADA’s suggestions [18]. Recently also the IDF and WHO pointed towards HbA1c as diagnostic tool for T2DM [19], recommending a cutoff point for the diagnosis of less then 6.5%. Despite the widespread use of HbA1c in clinical settings, different cutoff points were recommended for the diagnosis of T2DM, besides ≥6.5% from ADA and WHO [18,19] also ≥7% from other sources, such as the recommendation of Davidson and Schriger [20] based on reassessing 2,712 individuals ‘without diabetes history’ and the use of ≥7% by the New York City A1c Registry [21]. The distribution of HbA1c in a sample of 323 healthy Thai individuals determined the 2.5 and the 97.5 percentiles to be 2.9 to 4.9% respectively, with a mean VPG of 5.11 mmol/L (92.1 mg/dl) [22]. The ROC curve for HbA1c as a reference with a 7% cutoff point resulted in an optimal screening test of 5.03 mmol/L (90.1 mg/dl), which is similar to the above quoted findings. However, the associated specificity of 50% would be unacceptable for the clinical settings. Despite this the standard cutoff point set by the MoPH was a VPG of 7 mmol/L (126 mg/dl) and it was against the objective of the study to overrule this.

The somehow ‘low’ prevalence of chronic diseases usually results in a low PPV indicating the burden of the collaborating hospitals to check a high number of individuals with false positive results. In this situation a slight increase in prevalence results in a remarkable increase in PPV. That could have been achieved by selecting individuals with a high risk of having T2DM, such as elevating the cutoff point for age to 45 years and selecting only over nourished and obese individuals as well as those with a family history of T2DM for being eligible for screening. Again this was against the objective of the study since the MoPH only excluded individuals with an age of <35 years to be the target for screening. PPV also might increase due to lowering the cutoff point of the clinical reference, in this case from HbA1c values of ≥7% to ≥6.5% because the lower cutoff point will increase the prevalence and by this PPV. In fact a cutoff point of 6.5% HbA1c increases PPV for CBG to 67.9% and VPG to 70.4% (unpublished data).

Another important aspect however is to achieve an optimal sensitivity of the screening method. Using HbA1c ≥6.5% as cutoff point sensitivity for both screening indicators decreased, from 45.6% to 31.0% for CBG and from 39.7% to 28.4% for VPG (unpublished data). It might be argued that the low sensitivity by selecting 6.5% HbA1c as clinical reference is due to a high proportion of screened individuals actually not having the disease and consequently normal CBG and VPG values. The intention was to increase the probability that the clinical reference, here an HbA1c of 7% and above really identify a diseased person.

According to the results of this study, the determination by CBG is better than determination of VPG, however, the fact remains that the associated sensitivity of 45% for CBG results is rather low, and it is even lower for the VPG determinations.

Weighing the pros and cons of the use of HbA1c in so-called ‘*developing countries*’, the authors of a recent publication concluded that HbA1c should not be used for the diagnosis of T2DM in these settings [23]. The authors argued that the threshold for diagnosis, the cost of the test, the absence of a standardization network and the low sensitivity were all factors that make HbA1c an inappropriate tool for T2DM diagnosis. As far Thailand is concerned, HbA1c is often used in hospitals in monitoring T2DM in patients. Presently district and provincial hospitals have equipment and financial means to determine HbA1c correctly and each has a responsible laboratory or will be included into a standardization network. Therefore, a low sensitivity of CBG results in itself might not be a sufficient argument against the use of HbA1c as clinical reference in Thailand. Even in rural Thailand, villagers are well aware of at least the essentials of T2DM and know that blood glucose levels correlate to dietary intake [24].

An additional argument against the indiscriminate use of HbA1c is that the variation of the glycated hemoglobin also depends on HbA1c variants and ethnicity [25-28]. Other factors associated with plasma glucose levels have been reviewed recently by Herman [29] and Gomez-Perez et al. [23] and include anemia and iron deficiency as well as abnormal hemoglobins. Testing the applicability of HbA1c for Thai individuals as reported above [22] did not suggest that the Thai population was different from Caucasians as far as HbA1c levels are concerned. However, the individuals tested had been derived from a population in Bangkok and not from a population of the Northeast of the country, where anemia and iron deficiency are common and Thalassemia is a public health problem [30,31]. Haemoglobin E (Hb E) is the most common trait for hemoglobinopathies prevalent in the Northeast of Thailand [32]. Adjusted HbA1c values were higher in a group of Asians in comparison to white individuals with Impaired Glucose Tolerance (IGT) but the adjusted HbA1c difference was insignificant (5.78% versus 6% respectively) [29]. Erythrocytes are vulnerable in the Thalassemia disease [33] and the decrease of the mean erythrocyte age falsely lowers HbA1c test results [34]. Weak but significant differences between HbA1c levels of ‘normal’ subjects and hetero- as well as homo-zygote Hb E carriers have been determined previously [35]. However, the median values of HbA1c levels of the Hb E groups have been below the so called ‘normal’ controls. Therefore, the high number of false negative results when using HbA1c as clinical reference and fasting plasma glucose levels as screening test should not be due to the prevalence of thalassemia and Hb E.

An additional hint that false negative screening results against HbA1c might not be related to haematological issues but might be an indication of the VPG inadequacy as a screening tool is the result of a study undertaken some years ago, close to the area where this investigation has been based [11]. At that time (1995), HbA1c was not used as a reference, rather OGTT was preferred, with the cutoff point of 11.1 mmol/L (200 mg/dl), and the screening tool was fasting blood glucose, with a cutoff point of 7.8 mmol/L (140 mg/dl). These settings resulted in a sensitivity of 43.7%, specificity 89.8% and the PPV 37.8%. It seems that in the case of Thailand the use of both HbA1c and OGTT as final diagnostic tools results in similarly low sensitivities, as long as fasting blood glucose measurement is applied as screening tool.

Chronic kidney diseases may also affect HbA1c levels through increased blood urea nitrogen levels, causing the formation of carbamylated haemoglobin which cannot be differentiated in the determination from HbA1c [36]. In a recent publication from Thailand the proportion of T2DM patients with microalbuminuria was found to be about 40% [37]. Therefore, it cannot be excluded that some of the cases of undetected T2DM within the population screened for this study may have microalbuminuria. However, it is unlikely that undetected chronic kidney disease already developed to such an extent that it could have influenced the level of HbA1c.

It has been reported recently that HbA1c is insufficient to detect T2DM in the case of early diabetic status [38]. The present situation in Thailand mainly demands the identification of those who have been suffering from T2DM for quite some time already, so that this limitation of HbA1c at the present time may be overlooked.

Further investigations are necessary to recognize the reasons for the low sensitivity of the screening indicators. It has been reported recently that a high proportion of T2DM patients obviously do not feel comfortable to admit that they do not follow the advice of health personnel [39]. In general a Thai villager is quite well informed about the basics of diabetes mellitus [24]. It might well be that, in an ill attempt to have a favorable screening result, participants might limit energy and carbohydrate intake for a number of days before screening, since the event is arranged about a week in advance. The failure to identify individuals with yet undetected T2DM might be less a technical problem related to HbA1c, CBG or VGP considering that a similar investigation using OGTT as reference standard some years before resulted in a similarly insufficient VPG sensitivity [11]. The underlying reason for the observed discrepancies between VPG and a clinical standard, either being OGTT or HbA1c, might be due to the fact that the villagers can somehow influence the VPG, in reducing carbohydrate and calorie intake before screening, but not the latter tests. What the villagers may not be aware of is that HbA1c values reflect the status of glucose levels over approximately a three months period. In the context of Thai culture, villagers may be sensitive about being confronted by the health staff after being checked and found with unfavorable results. Health promotion experts might further investigate this phenomenon and try to change the attitude of the villagers.

## Conclusions

This study has shown that screening for T2DM using fasting capillary or plasma glucose levels determined by CBG and VPG, using HbA1c as the clinical reference, results in a very low sensitivity. This indicates that over 50% of diseased T2DM cases are not detected by such screening processes. Similar results were obtained by an earlier study using OGTT as the clinical reference [11]. More studies are necessary to explore reasons for this phenomenon. It is unrealistic to assume that OGTT will be used by the clinicians instead of HbA1c until all the possible implications have been sufficiently surveyed as suggested by Gomez-Perez et al. [23], even in middle-income countries such as Thailand.

## Competing interests

There are no competing interests.

## Authors’ contributions

BM, PaS, PoS and FPS contributed equally to planning, supervising, and evaluating the project, while WT, MT, and SW contributed equally to the field work. All authors read and approved the final manuscript.

## Pre-publication history

The pre-publication history for this paper can be accessed here:

http://www.biomedcentral.com/1471-2458/12/349/prepub
